# Mechanisms of Homeostatic Synaptic Plasticity *in vivo*

**DOI:** 10.3389/fncel.2019.00520

**Published:** 2019-12-03

**Authors:** Hey-Kyoung Lee, Alfredo Kirkwood

**Affiliations:** ^1^Department of Neuroscience, Mind/Brain Institute, Johns Hopkins University, Baltimore, MD, United States; ^2^Kavli Neuroscience Discovery Institute, Johns Hopkins University, Baltimore, MD, United States

**Keywords:** sliding threshold, metaplasticity, BCM theory, synaptic scaling, cortical plasticity, homeostasis, hebbian plasticity

## Abstract

Synapses undergo rapid activity-dependent plasticity to store information, which when left uncompensated can lead to destabilization of neural function. It has been well documented that homeostatic changes, which operate at a slower time scale, are required to maintain stability of neural networks. While there are many mechanisms that can endow homeostatic control, sliding threshold and synaptic scaling are unique in that they operate by providing homeostatic control of synaptic strength. The former mechanism operates by adjusting the threshold for synaptic plasticity, while the latter mechanism directly alters the gain of synapses. Both modes of homeostatic synaptic plasticity have been studied across various preparations from reduced *in vitro* systems, such as neuronal cultures, to *in vivo* intact circuitry. While most of the cellular and molecular mechanisms of homeostatic synaptic plasticity have been worked out using reduced preparations, there are unique challenges present in intact circuitry *in vivo*, which deserve further consideration. For example, in an intact circuit, neurons receive distinct set of inputs across their dendritic tree which carry unique information. Homeostatic synaptic plasticity *in vivo* needs to operate without compromising processing of these distinct set of inputs to preserve information processing while maintaining network stability. In this mini review, we will summarize unique features of *in vivo* homeostatic synaptic plasticity, and discuss how sliding threshold and synaptic scaling may act across different activity regimes to provide homeostasis.

## Introduction

**FIGURE 1:**
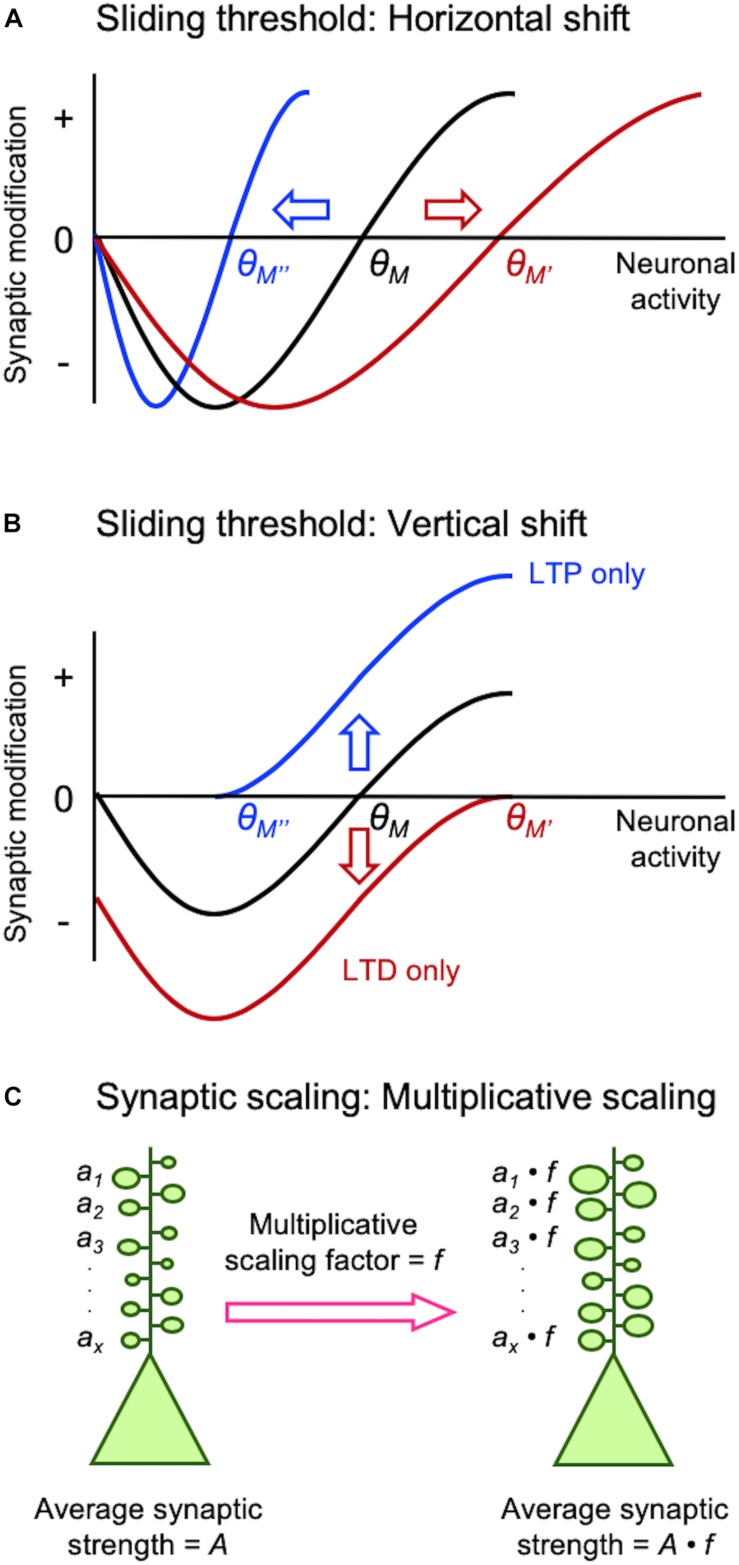
Different models of homeostatic synaptic plasticity comparison of sliding threshold model **(A,B)** and synaptic scaling **(C)**. Sliding threshold model posits that the synaptic modification threshold (θ*_*M*_*) changes as a function of past activity of a neuron. When integrated past activity is high θ*_*M*_* slides up to a higher value (θ*_*M*__’_*) promoting LTD, while with lower overall activity θ*_*M*_* slides down to a lower value (θ*_*M*__”_*) to preferential induce LTP. Expression of LTP or LTD as a consequence of sliding θ*_*M*_* acts to provide homeostasis of the average neural activity. θ*_*M*_* can slide via a horizontal shift **(A)**, which is implemented by altering the induction mechanisms of LTP/LTD such as regulation of GluN2B-containing NMDARs. θ*_*M*_* can also slide by a vertical shift **(B)**, which is mediated by changes in the expression mechanisms of LTP/LTD such as alteration in AMPAR phosphorylation state. Synaptic scaling was initially reported to occur globally across all synapses. A key feature that allows preservation of information stored at individual synapses despite global adjustment of synaptic weights is via multiplicative scaling **(C)**. Individual synaptic weights (*a*_1_…*a*_*x*_) are multiplied by a same scaling factor (*f*), which is greater than 1 for adapting to inactivity and less than 1 for adaptation to increased activity.

**FIGURE 2:**
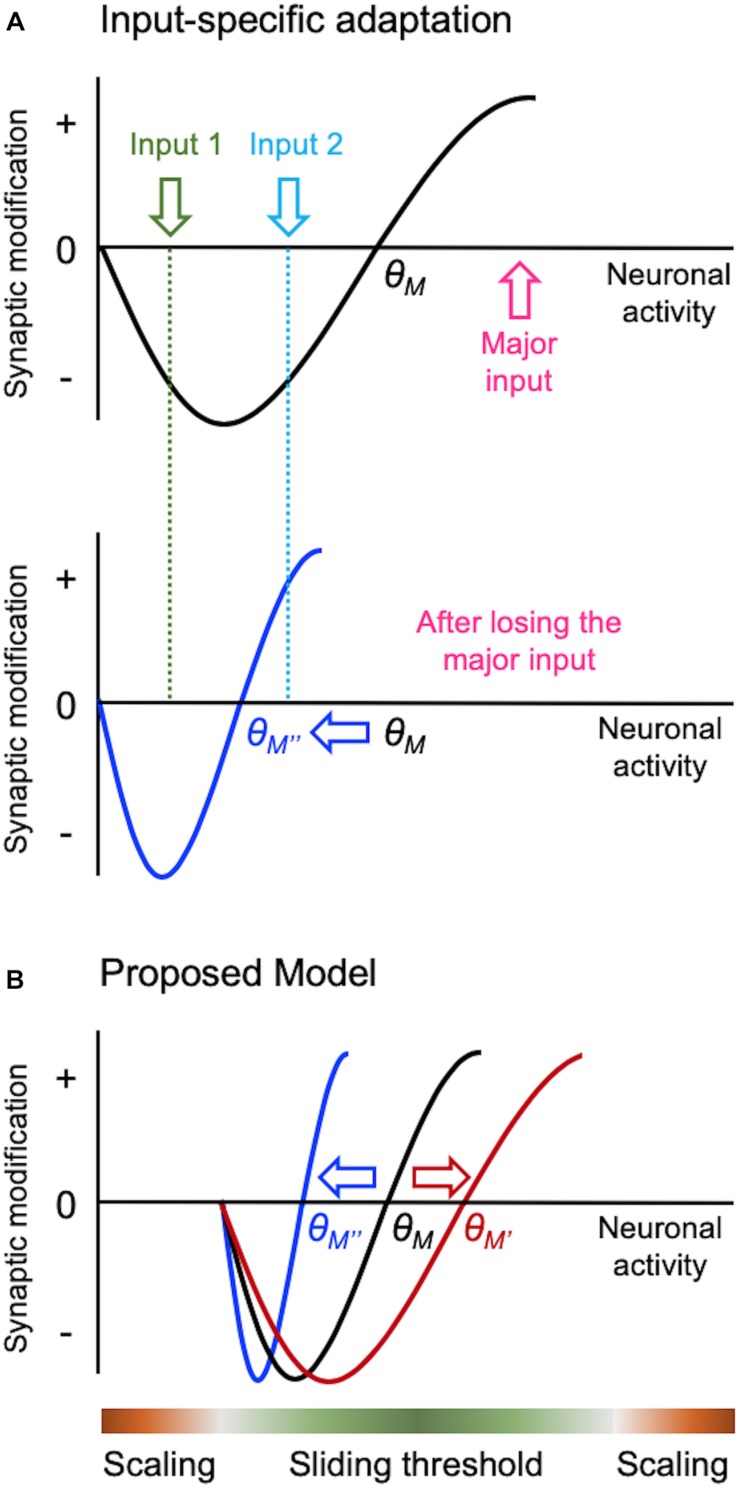
Input-specific homeostatic synaptic plasticity and distinct activity regime. There are specific considerations needed when implementing homeostatic regulation in intact circuits *in vivo*, such as a need to provide homeostasis in an input-specific manner. Sliding threshold model can easily accomplish input-specificity as depicted in panel **(A)**. When overall activity of a neuron is reduced, such as due to loss of its major input, θ*_*M*_* slides down. This causes previously weak Input 2 to cross the LTP threshold for synaptic potentiation, but leaves the less active input (Input 1) in the LTD range. Such input-specific adaptation allows the neuron to dynamically update its synaptic weights to process the most active input(s) in the context of its overall activity. We propose that sliding threshold and synaptic scaling operate across different activity regimes *in vivo* as shown in panel **(B)**. Based on the advantage sliding threshold endows intact neural networks, such as always adapting to the most relevant inputs as shown in panel **(A)**, we surmise that this is the dominant mode of homeostatic adaptation within most physiological range of activity. However, sliding threshold is less likely to be effect at providing homeostasis at extreme ranges of activity. For instance, when activity levels are too low, even if the θ*_*M*_* slides, there will be insufficient activity to activate NMDARs to drive potentiation of synapses. We suggest that NMDAR-independent synaptic scaling will be more effective at providing homeostatic adaptation with inactivity. At the other extreme, synaptic scaling will be much more effective at dampening overactive circuits, because it can globally reduce the strength of synapses.

A major challenge faced by neural circuits is to maintain proper neural processing while enabling effective information storage mediated by activity-dependent synaptic plasticity. This is not trivial, because plasticity of synaptic connections innately alters the flow of information between neurons. Furthermore, activity-dependent synaptic plasticity, namely long-term potentiation (LTP) and long-term depression (LTD), creates positive feedback which when uncompensated lead to network instability. In this mini review, we will compare two models of homeostatic synaptic plasticity, sliding threshold and synaptic scaling (Figure 1), and present emerging ideas as to how these two different models may interact to provide network stability *in vivo* (Figure 2).

Earlier studies on neural networks encountered difficulty in maintaining network function when solely engaging Hebbian synaptic plasticity for learning algorithms (discussed in Cooper and Bear, 2012). In one successful theory that allowed network stability developed by Leon Cooper’s group, the threshold for synaptic plasticity is controlled by integrated past neuronal activity (Bienenstock et al., 1982; Bear et al., 1987; Cooper and Bear, 2012). This theory termed the “sliding threshold” or “BCM model” not only explained development of neural feature selectivity and *in vivo* visual cortex plasticity, but it also made specific predictions that were experimentally verified subsequently (Bienenstock et al., 1982; Bear et al., 1987; Cooper and Bear, 2012). The key feature of this model is that the induction threshold for LTP and LTD is determined by past neural activity (Figures 1A,B). Specifically, a period of high activity increases the threshold for LTP induction, which meant most activity would fall below the synaptic modification threshold resulting in LTD. In theory, net LTD in the synaptic population should reduce neural activity even when other factors (e.g., inhibition and excitability) are unchanged. Prolonged low activity decreases the synaptic modification threshold to promote LTP across synapses. Experimental support for the sliding threshold model comes primarily from studies in sensory cortices, where sensory deprivation alters the synaptic modification threshold to favor LTP (Kirkwood et al., 1996; Hardingham et al., 2008; Guo et al., 2012).

Synaptic scaling is another popular model that provides homeostasis by adjusting the synaptic gain. While the sliding threshold model was initially proposed to explain the development of neural response selectivity and experience-dependent cortical plasticity, the premise of synaptic scaling was to explain stability of network activity propagation and firing rate homeostasis (Turrigiano and Nelson, 2004). In brief, prolonged inactivity leads to upscaling of excitatory synapses, while prolonged increase in activity downscales them to maintain overall average firing rate. Initial experimental support for synaptic scaling has come from *in vitro* neuronal culture models where activity was manipulated globally using pharmacological methods. Global inhibition of neural firing by application of tetrodotoxin (TTX) scales up excitatory synapses, while increasing neural activity by pharmacologically blocking inhibition scales down the strength of synapses (O’Brien et al., 1998; Turrigiano et al., 1998).

While both sliding threshold and synaptic scaling can provide similar homeostatic control by regulating synaptic strength, they differ in one key element. Sliding threshold model operates by altering the induction threshold for LTP/LTD, hence by nature requires neural activity to manifest the synaptic changes. Therefore, even if the synaptic modification threshold has changed based on integrated past activity, if there is insufficient neural activity through any of the synapses, there will be no change in synaptic gain. In contrast, synaptic scaling can occur without neural activity. Indeed, blocking all activity with TTX scales up excitatory synapses (O’Brien et al., 1998; Turrigiano et al., 1998). In addition, sliding threshold model posits that homeostatic control of synaptic strength will be input-specific even if the threshold is modified globally across the cell. This is because synapses that receive activity that falls below the synaptic modification threshold will undergo LTD, while those receiving activity surpassing the threshold will express LTP (Cooper and Bear, 2012). This is different from synaptic scaling where most synapses will show the same polarity of change in synaptic gain, unless the scale of operation is local as has been shown in some experimental preparations (reviewed in Turrigiano, 2008).

In the following sections, we will discuss evidence from *in vivo* preparations as to how each homeostatic synaptic plasticity model could operate, and provide evidence supporting a novel view that these two forms of homeostatic synaptic plasticity models likely operate under different activity regimes.

## Demonstration of Homeostatic Synaptic Plasticity *In Vivo*

Experience-dependent homeostatic synaptic plasticity has been demonstrated in various *in vivo* preparations (Whitt et al., 2014). The first experimental evidence came from studies on metaplasticity showing that prolonged visual deprivation alters the induction threshold for LTP/LTD (Kirkwood et al., 1995, 1996). Dark-rearing, expected to reduce the overall activity in visual cortex, decreased the induction threshold for LTP as predicted from the model (Figure 1A). Subsequent studies showed that the reduced LTP threshold resulted from an increased proportion of GluN2B-containing NMDARs at synapses (Quinlan et al., 1999; Philpot et al., 2001, 2003). GluN2B subunits have a longer current duration than GluN2A (Rumbaugh and Vicini, 1999), hence ideally suited to reduce the induction threshold for LTP. The opposite is also the case: increasing sensory experience reduces the proportion of synaptic GluN2B shifting the modification threshold to favor the induction of LTD (Quinlan et al., 1999). In parallel to sliding the induction threshold for synaptic modification, a later study demonstrated that metaplasticity can also manifest by alterations in the expression mechanisms of LTP/LTD (Huang et al., 2012). In particular, Huang et al. (2012) demonstrated that neuromodulators coupled to Gs-proteins are critical for LTP and will shift the synaptic modification function to produce an LTP-only state, while Gq-coupled neuromodulators produces an LTD-only state. This mode of metaplasticity shifts the synaptic modification curves vertically (Figure 1B), compared to lateral shifts produced by alterations in the induction mechanisms of LTP/LTD (Figure 1A). A unique aspect of this vertical shift in synaptic modification function by neuromodulators is that it puts synapses in LTP-only or LTD-only mode by changes in neuromodulatory tone coupled to internal states. Mechanistically, such vertical shift in synaptic modification function is brought about by changes in the expression mechanisms of LTP/LTD, which relates to the phosphorylation state of AMPARs (Seol et al., 2007). In particular, phosphorylation serine-845 (S845) residue on the GluA1 subunit of AMPARs is necessary for both LTP promoted by Gs-coupled neuromodulators and LTD promoted by Gq-coupled neuromodulators, while GluA1 serine-831 (S831) is necessary only for Gq-coupled neuromodulator induced LTD (Seol et al., 2007).

Visual cortex has also been a model used to demonstrate synaptic scaling *in vivo*. For example, visual deprivation in the forms of intraocular injection of tetrodotoxin (TTX) (Desai et al., 2002), dark exposure (Goel et al., 2006, 2011; Goel and Lee, 2007; Gao et al., 2010; He et al., 2012; Petrus and Lee, 2014), dark-rearing (Goel et al., 2006), enucleation (He et al., 2012; Barnes et al., 2017), or retinal lesions (Keck et al., 2013) scales up mEPSCs. Interestingly, in V1 upscaling of mEPSCs has layer specific sequential critical periods, where layer 4 (L4) ends by postnatal day 21(P21) (Desai et al., 2002) while in layers 2/3 (L2/3) it starts by P21 and persist through adulthood (Goel and Lee, 2007). The rates of scaling up and down are asymmetric. It takes at least 2 days of darkness to upscale mEPSCs (Goel and Lee, 2007), but only 2 h of light re-exposure to fully reverse it (Gao et al., 2010), suggesting different temporal integration for each process. Experience-dependent synaptic scaling has been reported in other sensory cortices besides V1: in L2/3 of auditory cortex after sensorineural hearing loss (Kotak et al., 2005) or conductive hearing loss (Teichert et al., 2017), in L4 of barrel cortex after afferent nerve (i.e., infraorbital nerve) transection (Yu et al., 2012), but not in L2/3 of barrel cortex after whisker plucking (Bender et al., 2006; He et al., 2012; Li et al., 2014) (but see Glazewski et al., 2017). This intriguing absence of synaptic scaling with whisker plucking will be discussed in section “Specific Challenges Of Homeostatic Synaptic Plasticity *in vivo*.”

Mechanistically, scaling up and down are not the reverse of each other, but rely on distinct molecular signaling. In V1, upscaling of mEPSCs after DE correlates with phosphorylation of GluA1 on S845, synaptic appearance of Ca^2+^-permeable AMPARs (Goel et al., 2006), and mGluR1 (Chokshi et al., 2019), while downscaling is dependent on Arc (Gao et al., 2010), mGluR5, and Homer1a (Chokshi et al., 2019). Although GluA1-S845 is necessary for upscaling, it alone is not sufficient to recapitulate multiplicative scaling (Goel et al., 2011). Multiplicative change is a key feature of synaptic scaling (Figure 1C), because it preserves information stored as different weights across synapses in a neuron (Turrigiano et al., 1998). However, multiplicative scaling is only observed early in development (P21 to ∼P35) in V1 (Goel and Lee, 2007). We interpreted this to suggest that synaptic scaling in adults is not global, but limited to a subset of synapses. Consistent with this interpretation, we reported that DE-induced upscaling of mEPSCs reflects potentiation of lateral intracortical (IC) synapses, but feedforward (FF) synapses from L4 to L2/3 are immune to this type of plasticity (Petrus et al., 2015). Similarly, downscaling of mEPSCs with visual experience is also limited to IC synapses (Chokshi et al., 2019). Such input-specific synaptic scaling is observed in L5 of V1 at the level of dendritic spine plasticity. It was reported that visual deprivation via enucleation leads to enlargement of dendritic spines on L5 neurons, which was specific to dendritic branches with recent spine loss (Barnes et al., 2017). Based on these new observations showing that sensory experience-dependent homeostatic plasticity of mEPSCs is input-specific and also other recent evidence discussed below, we propose that the apparent synaptic scaling induced *in vivo* with sensory manipulations is actually a manifestation of sliding threshold metaplasticity see section “Different Activity Regime May Recruit Distinct Homeostatic Synaptic Plasticity *In vivo*.”

## Specific Challenges of Homeostatic Synaptic Plasticity *In Vivo*

One of the challenges of homeostatic plasticity operating *in vivo* is that not all inputs are identical. Cortical neurons receive diverse set of inputs from multiple sources. For example, V1 not only receives inputs from the primary visual thalamus (dLGN), but also from other sensory areas (Lakatos et al., 2007; Iurilli et al., 2012; Yoshitake et al., 2013; Ibrahim et al., 2016), subcortical areas (Roth et al., 2016), higher visual areas (Coogan and Burkhalter, 1993; Dong et al., 2004; Ji et al., 2015; Marques et al., 2018), and other cortical areas (Wall et al., 2016). Input diversity is not a particular property of V1, but rather a general property of highly interconnected cortical networks. It is inconceivable then that all of the inputs are equivalent and share the same levels of input activity. Therefore, homeostatic synaptic plasticity needs to occur in a way to preserve information storage and processing capacity of a diverse set of networks in which a particular neuron participates in. It was proposed based on computational modeling that input-specific homeostatic plasticity is much better suited to improve information processing than global synaptic scaling (Barnes et al., 2017) (for further discussions see Keck et al., 2017). In this particular study, the unit of homeostatic control was proposed to be a dendritic branch. There are several observations that similar inputs tend to cluster on the same dendritic branch (Wilson et al., 2016; Iacaruso et al., 2017), thus branch-specific homeostatic adaptation would allow functional input-specific control that is independent from each other.

Another unique challenge to study *in vivo* homeostatic plasticity is that not all sensory manipulations lead to the same changes. As mentioned above, in the case of visual deprivation, majority of the paradigms ranging from intraocular TTX injection, dark-rearing, dark-exposure, enucleation, and retinal lesions scales up mEPSCs in V1 (Desai et al., 2002; Goel et al., 2006; Goel and Lee, 2007; He et al., 2012; Keck et al., 2013; Barnes et al., 2017). However, lid suture typically do not (Maffei and Turrigiano, 2008; He et al., 2012; Bridi et al., 2018) (but see Hengen et al., 2013). Similarly, in the barrel cortex afferent nerve transection upregulates mEPSCs (Yu et al., 2012; Chung et al., 2017), but not whisker deprivation (Bender et al., 2006; He et al., 2012; Li et al., 2014); but see Glazewski et al. (2017). Differences in outcome may stem from the degree of activity changes associated with various sensory manipulations. In the visual deprivation cases, dark-rearing or dark-exposure removes vision, but leaves spontaneous activity in the retina and through the visual pathway. Recently, we reported that dark-exposure for a few days lead to increase in spontaneous firing of V1 neurons (Bridi et al., 2018). Intraocular TTX injection and enucleation removes vision and spontaneous activity in the retina, but it has been noted that dLGN neurons undergo oscillatory activity (Linden et al., 2009). Lid suture is a much milder form of deprivation where form vision is largely lost, but vision is not totally abolished. Visual stimulation seen through the closed eyelids can elicit small but measurable visually evoked potentials (VEPs) in V1 (Blais et al., 2008). As exemplified, the level of sensory deprivation and the consequent changes in neural activity through the sensory pathway is not identical across different paradigms. This is not likely just limited to the visual system, but it extends to other sensory cortices. For example, the reason that whisker deprivation in most cases fails to induce changes in mEPSCs in barrel cortex L2/3 (Bender et al., 2006; He et al., 2012; Li et al., 2014) may be because it is similar to lid suture where afferent activity is not completely abolished. In any case, study of homeostatic plasticity *in vivo* will need to be interpreted in the framework of the specific type of manipulation done, which adds complication compared to pharmacological manipulation of activity that can be achieved *in vitro*.

Further complications when studying intact cortical circuits is that one needs to consider the specific cell-type and lamina that is being investigated. One reason is that different laminae exhibit distinct critical period for plasticity with L4 typically showing early plasticity followed by opening of plasticity in L2/3 (Desai et al., 2002; Goel and Lee, 2007; Jiang et al., 2007). Also the means in which different laminar neurons adapt to the same types of sensory manipulations are quite distinct (reviewed in Whitt et al., 2014; also see Glazewski et al., 2017). Even within the same layer, cell type also seems to matter. For example, in L5 of barrel cortex, there is distinct plasticity triggered by changes in sensory experience based on specific cell-types (Greenhill et al., 2015; Glazewski et al., 2017). Ultimately, there will be differences in input activity based on the different functional circuit in which a particular neuron is part of. Hence, it is not surprising that different neurons would respond differently to a particular *in vivo* manipulation.

## Different Activity Regime May Recruit Distinct Homeostatic Synaptic Plasticity *In Vivo*

There is emerging evidence that different activity regimes may recruit distinct modes of homeostatic adaptation *in vivo* (Figure 2B). Bridi et al. reported that visual deprivation leads to metaplasticity mode of homeostatic adaptation in V1, but silencing cortical activity more by pharmacologically increasing tonic inhibition produces synaptic scaling-like adaptation (Bridi et al., 2018). Of interest is that visual deprivation-induced metaplasticity is likely driven by increased spontaneous activity acting on GluN2B-containing NMDARs. This counters the conventional notion that sensory deprivation leads to loss of activity in the corresponding sensory cortex, and that inactivity is driving homeostatic adaptation. This work suggests that sensory deprivation-induced homeostatic plasticity requires activity, for instance, in the form of elevated spontaneous activity. We also recently reported that dark-exposure induced upscaling of mEPSCs in V1 L2/3 is dependent on NMDAR activity (Rodriguez et al., 2019), which further corroborates the involvement of sliding threshold that acts on NMDAR-dependent LTP/LTD processes. Our current working model is that sensory deprivation-induced reduction in synaptic modification threshold coupled with increased spontaneous activity potentiates synapses to mediate homeostatic increase in excitatory synaptic gain. Increased spontaneous activity has been reported in A1 with auditory deprivation (Kotak et al., 2005), and infraorbital nerve transection that potentiates synapses in barrel cortex also increases GluN2B-containing NMDARs (Chung et al., 2017). These findings suggest that similar mechanism may operate across sensory cortices.

Sliding threshold mediated homeostatic adaptation has an advantage that it can easily implement input-specificity (Figure 2A). Inputs that exhibit activity above the threshold will produce potentiation, those falling below will depress, and inputs with minimal activity or activity at the threshold will not change. Such input-specific homeostatic adaptation has one advantage in that it will allow the circuit to preferentially process currently active inputs despite overall activity changes. Therefore, the cortical networks can be dynamically reconfigured for processing the most relevant information in the context of overall activity in the circuit. It is of interest to note that input-specific homeostatic plasticity is more prevalent in mature cortex (Goel and Lee, 2007; Ranson et al., 2012; Petrus et al., 2015; Barnes et al., 2017; Chokshi et al., 2019).

While sliding threshold provides homeostasis with sensory manipulation paradigms, synaptic scaling seems to also be present *in vivo* but at extreme activity ranges (Figure 2B). For example, reducing cortical activity by pharmacologically increasing tonic inhibition leads to upscaling of mEPSCs, which is not dependent on NMDARs (Bridi et al., 2018). We surmise that synaptic scaling may also operate when neural activity is increased to an extreme level. The rationale is that under either extreme activity regimes sliding threshold may not be effective. For example, under extremely low activity even if the synaptic modification threshold slides down, there may not be sufficient level of activity to drive LTP. Therefore, NMDAR-independent plasticity, such as synaptic scaling, may be better suited for synaptic adjustments under this condition. Similarly, when there is extremely high neural activity across all inputs, as would occur during seizures, having input-independent global synaptic scaling is likely a more efficient way to dampen activity.

## Conclusion

We summarized the specific challenges met when homeostatic plasticity operates in intact circuits *in vivo* with diverse sets of inputs. We propose that sliding threshold operates across activity ranges that can recruit NMDAR-dependent input-specific synaptic plasticity to maintain optimal processing of most relevant information despite overall changes in activity, while synaptic scaling may operate at extreme activity ranges to act as a failsafe.

## Author Contributions

Both authors listed have made a substantial, direct and intellectual contribution to the work, and approved it for publication.

## Conflict of Interest

The authors declare that the research was conducted in the absence of any commercial or financial relationships that could be construed as a potential conflict of interest.
